# Exploring care-experienced young people’s perceptions of the use of administrative data in research

**DOI:** 10.23889/ijpds.v9i5.2529

**Published:** 2024-09-18

**Authors:** Aimee Cummings

**Affiliations:** 1 Cardiff University

## Objective and Approach

Beginning in 2018, a provincially-funded custodial housing model in Ontario, Canada transitioned to a supported housing model that allowed more autonomy for clients who experience severe and persistent mental illness (SPMI). The objective of this study was to compare health service use before and after transition to the new Community Homes for Opportunity (CHO) program in southwestern Ontario between 2017 and 2019. Information about clients who transitioned to the CHO program were obtained from the Ontario Ministry of Health and Long-Term Care and linked to health administrative data at ICES. Rates of emergency department (ED) visits, primary care visits, and specialist visits in the one year before and after implementation of the CHO program were compared using conditional Poisson models.

## Results

The following questions will be explored:

- What is understood by the terms ‘administrative data’ and ‘data linkage’?
- What are young people’s thoughts on how their data is used?
- What would they like to know about how their data is being used?
- WCan they think of reasons why you would not want their data to be used?
- Do they think that the data that is collected (from a social worker, GP, etc.) accurately reflects their experiences?

As well as this, important aspects of the collection of administrative data will be discussed.

## Conclusions

The output of this piece of work will be an infographic. The aim is that this infographic will contain two facets. First, it will illustrate how administrative data is collected and used (including a life cycle of the data). Second, it will contain information that young people regard as pertinent to this topic and will contain quotes ‘for young people, for young people’.

## Implications

The infographic from this work can be used by other researchers as part of their own public engagement work with young people.

